# Vitamin B12 and estradiol benzoate improve memory retrieval through activation of the hippocampal AKT, BDNF, and CREB proteins in a rat model of multiple sclerosis

**DOI:** 10.22038/IJBMS.2021.51469.11681

**Published:** 2021-02

**Authors:** Narjes Taherian, Gholamhassan Vaezi, Ali Neamati, Leila Etemad, Vida Hojjati, Mahmoud Gorji-Valokola

**Affiliations:** 1 Department of Biology, Damghan Branch, Islamic Azad University, Damghan, Iran; 2 Department of Biology, Mashhad Branch, Islamic Azad University, Mashhad, Iran; 3 Medical Toxicology Research Center, Mashhad University of Medical Sciences, Mashhad, Iran; 4 Department of Pharmacodynamics and Toxicology, School of Pharmacy, Mashhad University of Medical Sciences, Mashhad, Iran

**Keywords:** Estradiol benzoate, Hippocampus, Memory, Multiple sclerosis, Vitamin B12

## Abstract

**Objective(s):**

Multiple sclerosis (MS) causes extensive damage in the hippocampus. Vitamin B12 (vit B12) and estradiol benzoate (EB) have anti-inflammatory and re-myelination properties that make them proper in improvement of cognitive impairment. This study aimed to evaluate the effects of these compounds on learning and memory disturbances.

**Materials and Methods:**

77 adult male rats were implanted with stainless steel guide cannula bilaterally into the hippocampal area. The animals received 3 μl intrahippocampal EtB 0.01% and were randomly divided into eleven groups (7 rats/group). The groups included control, peanut oil (sham1), distilled water (sham 2), vit B12 (0.25, 0.5, 1 mg/kg), EB (25 and 50 mg/kg), vit B12 (0.25 mg/kg) plus EB (25 mg/kg), vit B12 (0.5 mg/kg) plus EB (25 mg/kg), and vit B12 (1 mg/kg) plus EB (50 mg/kg). The control group received intrahippocampal saline (as solvent). The locomotor activity and learning and memory functions were evaluated by open-field and shuttle-box tests, respectively. AKT, CREB, and BDNF levels were analyzed by Western blotting.

**Results:**

This study has found significant deficit in passive avoidance learning, locomotor activity, as well as decrease in the levels of phosphorylated AKT, BDNF, and CREB in groups that received EtB. Vit B12 (1 mg/kg), EB (50 mg/kg), and their combination markedly improved these side effects.

**Conclusion:**

This study demonstrated that vit B12 and estradiol benzoate, especially in combination therapy, can be helpful in treatment of memory problems and MS-induced dysfunction through activation of the hippocampal AKT, BDNF, and CREB proteins.

## Introduction

Multiple sclerosis (MS) is a multifocal inflammatory and demyelinating disorder that grossly affects the central nervous system (CNS) resulting in progressive neurological impairment. MS causes extensive damage in the brain and spinal cord (1), including brain volume reduction, ventricular enlargement, and spinal cord atrophy (2). Approximately 2.5 million people around the world suffer from MS as the most debilitating neurological disease after trauma. MS is induced by various factors, such as immune disorders, viral infections, genetics, environmental factors (3), and metals such as iron (4). In fact, the immune system acts against the axonal myelin sheath and causes chronic disability (5).

Based on previous study, the most important symptom of MS is myelin destruction, especially the gray matter atrophy (6), which is associated with a patient’s permanent disability (7). Oligodendrocyte precursor cells (OPCs), which are present in brain’s white matter and around MS lesions, are responsible for axonal re-myelination (8). Although endogenous re-myelination which may occur in MS patients, it is a relatively short-term process, imperfect, and ineffective (8). It is also indicated that a decrease in activity of the antioxidant enzymes, such as glutathione peroxidase and catalase, play an important role in MS disease induction (9).

Animal models of MS are so important to evaluate the pathological mechanisms of MS and how they may be targeted for therapeutic intervention. Experimental autoimmune encephalomyelitis (EAE) is the most widely used animal model for MS and induced by injection of proteins, myelin peptides, and other substances, such as EtB, in mice, rats and monkeys (10). Direct injection of EtB into the brain is a simple and repeatable model that can offer the advantage of evaluating neuronal demyelination, re-myelination, and remodeling processes in the CNS (11).

The hippocampus is composed of several sub-regions with different histology and functional characteristics that make them susceptible to various diseases (12). One of the most important sites of injury in MS and EAE is hippocampus which plays a major role in memory formation and retrieval. Cognitive deficits, including impairment of information processing speed, attention, and learning and memory formation, are presented at more than 50% of MS patients (13).

Currently, disease-modifying therapies for MS are focusing on decreasing the inflammatory response and the severity of clinical symptoms. The main goal of MS treatment is to identify new drugs that enhance oligodendrocyte differentiation and myelin sheath formation as well as minimize axonal degeneration and inflammation, immune system activity, and the rate and degree of disease progression. With regard to immunomodulatory, anti-inflammatory, and neuroprotective effects of vit B12 and EB, they can be considered as a possible candidates for the treatment of MS (14). 

Vitamin B12 (cobalamin) is a metal-organic compound the human body cannot synthesize and should be provided through diet, especially animal products, including meat and dairy products. The minimum human daily requirement for vit B12 is approximately 2.5 micrograms (15). Vit B12 absorption relies on two important factors, including intrinsic factor (aglycoprotein produced by the gastric parietal cells) and trans-cobalamin receptor (located in the mucosal cells of the ileum) (15). Vit B12, as a co-factor, plays a vital role in many body processes, such as hematopoiesis and myelin sheath production in the nervous system. Vit B12 deficiency induces inflammatory and neurodegenerative disorders, specially myelin sheath damage in the central and peripheral nervous systems (16).

Estriol, estradiol, and estrone are three main endogenous estrogens that exert their genomic and non-genomic effects through intracytoplasmic or nuclear receptors (ERα and β) and plasma membrane receptors (such as ER-X). The ERα and β have anti-inflammatory and neuroprotective effects and are located in the subgranular zone (SGZ) of the hippocampal dentate gyrus (17). Estradiol (18) and estriol can influence hippocampal neurogenesis (19) and have neuroprotective effects on EAE via the ERα receptor. In addition, several studies have indicated that the effect of estrogens and estrogen receptor (ER) agonists on diverse signaling pathways, resulted in proliferation and differentiation of oligodendrocyte cells (20, 21). Estrogen treatment meaningfully diminishes the severity of EAE in numerous strains of mice and also decreases inflammatory immune response (19, 22) by inhibiting pro-inflammatory cytokines, chemokines, extracellular matrix proteins, antigens, and dendritic cell function (18, 22). Estrogen treatment also reduces white (23) and gray matter (24) inflammation, demyelination, and axonal destruction in the spinal cord of EAE (23).

Based on the aforementioned research background, this study was designed to investigate the effects of vit B12 and EB alone or in combination on memory function in a rat model of MS.

## Materials and Methods

***Chemicals***

Vitamin B12 (cobalamin) (CAS # V2876), peanut oil (CAS # P2144), and estradiol benzoate (CAS # E8515) were purchased from Sigma-Aldrich (Germany). Ketamine hydrochloride and xylazine hydrochloride were provided by Merck (Rotexmeica, Germany).

***Animals***

In this experimental study, 77 adult male Wistar rats with an approximate weight of 230 to 250 g (two-month-old) were provided by the Animal Center, Department of Biology, Damghan Branch, Islamic Azad University, Damghan, Iran. All rats were maintained in standard conditions with a 12-hr light/dark cycle and at a temperature of 23±1 °C, with free access to food and water. Each rat was used only once. All behavioral experiments were performed in the light cycle between 9:00 a.m. to 2:00 p.m. These conditions were constantly maintained during the experimental period and all animal experiment protocols were performed in accordance with Ethical Committee Acts of Damghan Branch, Islamic Azad University, Damghan, Iran (IR.DB, IAU.REC.1397.628).

***Treatment***

**Figure 1:**
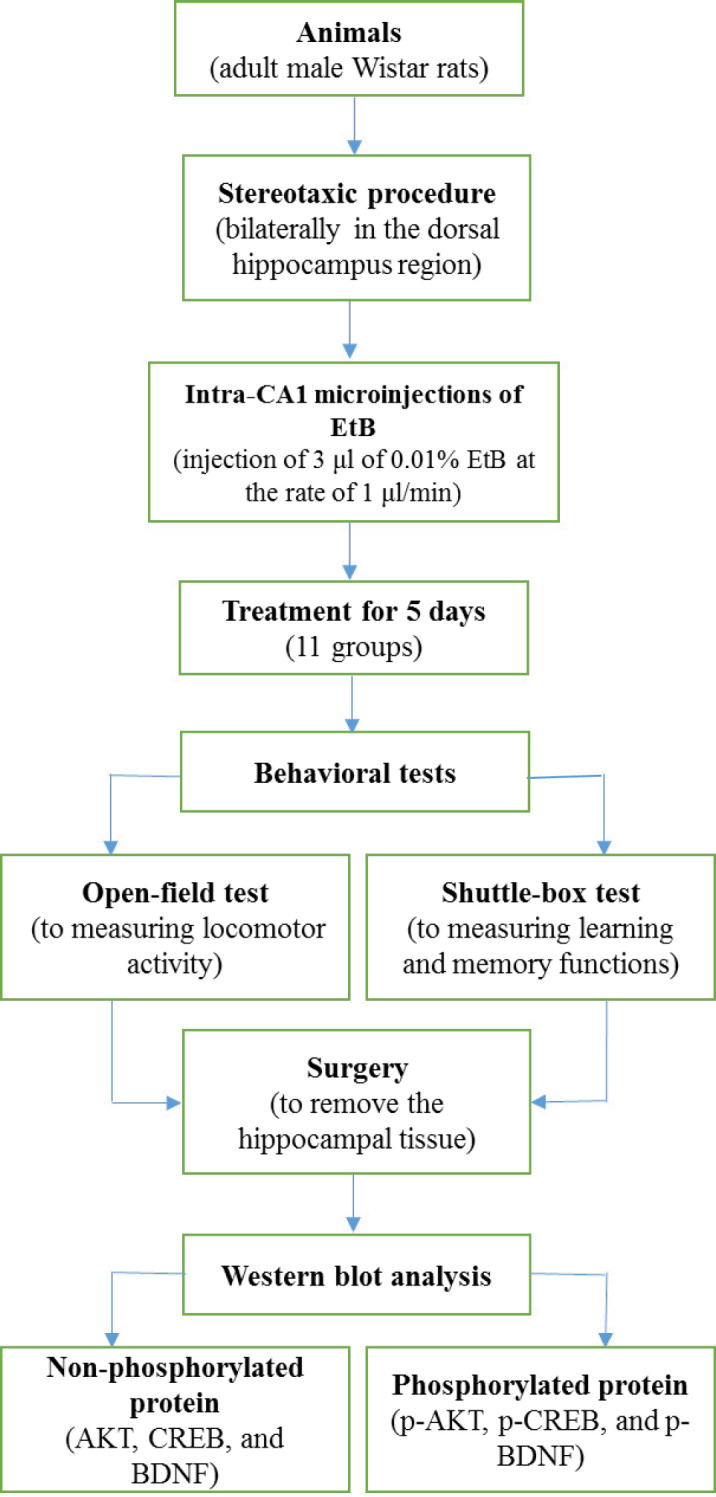
A schematic diagram of the experimental study design

All rats were anesthetized with IP injection of ketamine and xylazine at dose of 50 and 5 mg/kg, respectively. Anesthetized rats were placed on stereotaxic instrument in the skull-flat position for implantation of stainless steel guide cannula into the left and right hippocampal CA1 areas. After a recovery period of 5–7 days, 3 μl of 0.01% EtB in sterile 0.9% saline at the rate of 1 μl/min was injected into the hippocampal CA1 area (25). Three days after injection, the rats were divided into 11 groups and each group consisted of 7 rats, including control, sham 1 (peanut oil), sham 2 (distilled water), groups 4-6 (vit B12 at doses of 0.25, 0.5, 1 mg/kg), groups 7 and 8 (EB at doses of 25 and 50 mg/kg), group 9 (vit B12 (0.25 mg/kg) plus EB (25 mg/kg)), group 10 (vit B12 (0.5 mg/kg) plus EB (25 mg/kg)), and group 11 (vit B12 (1 mg/kg) plus EB (50 mg/kg)). The control group received intra-hippocampal normal saline (as solvent). Distilled water (as vit B12 vehicle) and vit B12 were gavaged while peanut oil (as EB vehicle) and EB were administrated by intraperitoneal (IP) injection. Treatment duration was 5 days. At the end of the period, the behavioral tests, including open-field for locomotor activity and shuttle-box for learning and memory function, were evaluated. Then, the animals were anaesthetized via IP injection of ketamine and xylazine at doses of 50 and 5 mg/kg, respectively and the hippocampus tissues were removed and stored at -80 °C until protein extraction was performed. A schematic diagram showing the experimental study design is exhibited in Figure 1. 

***Stereotaxic procedure***

The rats were anesthetized as mentioned above and then placed in a stereotaxic apparatus in a flat skull position (incisor bar±3.3 mm and ear bars positioned symmetrically) (Stoelting Co., Illinois, USA). Subsequently, the scalp was shaved and sterilized and the skin incised on the mid line. In the next step, two holes were drilled in the skull and two stainless steel guide cannulas (22 gauge, 12 mm length, and 0.7 mm diameter) were implanted bilaterally, 1 mm above the dorsal hippocampus (CA-1) (coordinates: anteroposterior (AP) = -2 mm from the bregma point, media lateral (ML) = ±1.6 mm from the sagittal suture on both sides, and dorsoventrally (DV) = -1.5 mm from the skull surface), according to rat brain atlas (Paxinos and Watson) (26). The position of guide cannulas and jewelers’ screws, for more stability, were permanently fixed to the bone using acrylic dental cement. The stainless steel insect pins in the size of 27-gauge were entered into the guide cannula to reduce the incidence of occlusion. Finally, all rats were allowed 5–7 days for recovery (27).

***Intra-MS/CA1 microinjections of drug***

For drug microinjection, all rats were quietly restrained by hand, the stainless steel stylets removed from the guide cannula, and switched with dental 27-gauge injection needles (1 mm below the tip of the guide cannula). Next, the needle was connected to a 10-µl Hamilton micro syringes by a polyethylene tube (PE-20). Injection of 3 μl of 0.01% EtB in sterile 0.9% saline at the rate of 1 μl/min was bilaterally performed into the hippocampal CA1 region (25). During the injection period, the animal was freely allowed to move without any stress. The drugs were injected 5–7 days after surgery and carried out 5 min before behavioral evaluation (27).

***Passive avoidance training test***

The passive avoidance test (PAT) is used to evaluate fear learning and memory in animal models of neurological disorders and subjects learning to avoid an unpleasant stimulus (such as a foot-shock). In this study, the PAT protocol was used according to Yousefi *et al*., 2012 (28) with little modification. Briefly, the PAT apparatus consisted of two compartments, including a light and a dark box (opaque resin, 20 cm × 20 cm × 30 cm) with a connecting open door (7 cm × 9 cm). A parallel stainless steel grid at 2.5 mm diameter and 1 cm intervals was located on the floor of the dark box to generate foot shock (29). In the first step, habituation phase, rats were allowed to stay in the experimental room for 1 hr. In the next step, acquisition trial or training stage, animals were gently placed in the white box, the guillotine lid was opened, and the animals were allowed to enter the dark box where the single electric shock (50 Hz, 3 sec, and 1 mA intensity) (Borj Sanat Co, Tehran, Iran) is done on the animal’s foot by an electrical generating device. Finally, the last step or the retrieval stage, was performed 24 hr after the training stage and during this phase the interval time between animal movements from light box into the dark box (step-through latency) was measured. The cut-off time of this step was 300 sec. All steps were manually implemented between 8:00 a.m. to 2:00 p.m.

***Open-field test***

The open-field test is carried out to measure behavioral responses, including locomotor activity, hyperactivity, and stereotypical and exploratory behaviors. Summary, each rat was placed in the center of the cage made of white wood (45 cm × 45 cm × 45 cm). The animal’s movements were recorded using a digital camera for 5 min. The locomotor activity was evaluated by measuring the number of peripheral (those adjacent to the walls), central, and total square crossings; the three measures were referred to as peripheral (PL), central (CL), and total (TL) locomotion, respectively. At the end of each experiment, the open-field device was cleaned using a damp sponge and a dry paper towel (30).

***Western blot analysis***

The hippocampal region of the rat brain tissue was rapidly separated, frozen in liquid nitrogen, and kept at -80 °C. Protein analysis of protein kinase B (AKT), cAMP response element-binding protein (CREB), and brain-derived neurotrophic factor (BDNF), as well as their phosphorylation types were performed by Western blotting assays on protein extraction from hippocampus tissues. Approximately, 200 mg of each sample was homogenized in lysis buffer, including 50 mM Tris-HCL with pH 7.4, 2 mM EDTA, 2 mM EGTA, 10 mM NaF, 1 mM sodium orthovanadate (Na3VO4), 10 mM β-glycerol-phosphate, 0.2% W/V sodium deoxycholate, 1 mM phenyl methyl sulfonyl fluoride (PMSF), and protease inhibitor cocktail (1% protease and phosphatase inhibitor cocktail) (Sigma, P8340) by a Polytron homogenizer(POLYTRON-PT10-35, Kinematica, Switzerland) in ice and next centrifuged at 10,000 g for 10 min at 4° C (31, 32). Subsequently, supernatants were collected and the concentration of total protein, via the Bradford protein assay kit (Bio-Rad), was determined. Then, protein lysate aliquots were instantly denatured at 95 °C in sodium dodecyl sulfate (SDS)-Laemmli sample buffer, 2 mM DTT for 5 min, and stored at -80 °C. In summary, equal quantities of proteins (50 μg) were separated through 12% SDS-polyacrylamide gel (SDS-PAGE gel) and transferred to polyvinylidene difluoride (PVDF) membranes (Millipore). The PVDF membranes were blocked with 5% skim milk or %1 bovine serum albumin (BSA) in Tris-buffered saline plus 0.1% Tween 20 (TBST) for 2 hr at room temperature to prevent non-specific protein binding. The membranes were incubated at 37 °C for 2 hr with primary rabbit monoclonal antibodies from Cell Signaling Technology (Beverly, MA, USA): anti-CREB (#4820), anti-BDNF (#3897), anti-AKT (#9272), anti-p-CREB (#9198), anti-p-BDNF (#47808), and anti-p-AKT (#9271) at a concentration of 1:1000 in TBST. After washing the membranes three times with TBST buffer (3×5 min in TBST), they were exposed with the secondary antibodies (anti-rabbit IgG) conjugated with horseradish peroxidase enzyme (HRP) (cell signaling, #7074) 1:3000 in TBST for 90 min at room temperature. After washing three times again, PVDF membranes were separately exposed to enhanced chemiluminescence (ECL) reagent (Pierce ECL Western Blotting Substrate) and H_2_O_2_ for 3 min. The intensity of protein bands was analyzed by Alliance 4.7 Gel Doc (UK). The protein expression levels were normalized versus beta-actin (#4967) protein level.

***Statistical analysis***

All data are presented as mean±standard deviation (mean±SD) (n=7). The statistical analysis of mean values was done by one-way ANOVA followed by the Tukey-Kramer test using SPSS (ver. 16.0) software. *P*˂0.05 was regarded as statistically significant.

## Results

***Effect of vitamin B12 and EB on passive avoidance learning and memory in EtB-induced multiple sclerosis***

**Figure 2:**
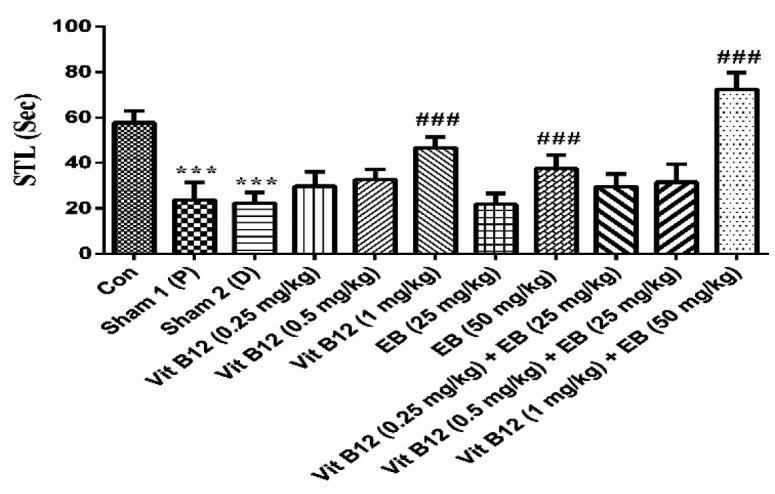
Effects of vit B12 (0.25, 0.5, and 1 mg/kg) and EB (25 and 50 mg/kg) alone or in combination therapy in passive avoidance test. Animals received EtB in sterile 0.9% saline at the rate of 1 μl/min into the hippocampal CA1 area. Three days after injection, the rats were randomly divided into 11 groups. Distilled water (as vit B12 vehicle) and vit B12 were gavaged and peanut oil (as EB vehicle) and EB were administrated by intraperitoneal injection. The control group received intra-hippocampal saline (as solvent). Treatment duration was 5 days. Data are shown as mean±SD, one-way ANOVA and Tukey–Kramer. ^***^P<0.001 compared with the control group, and^ ###^*P*<0.001 compared with the sham groups

As shown in Figure 2, there was a significant difference in step through latency (STL) between the control and sham (lesion) groups (*P*<0.001). Vit B12 at a dose of 1 mg/kg and EB at a dose 50 mg/kg alone or in combination therapy markedly increased STL and promoted fear learning and memory in comparison with sham groups (F (10, 66) = 49.58 and *P*<0.001).

***Effect of vitamin B12 and EB on locomotor activity in EtB-induced multiple sclerosis***

According to the results obtained from locomotor activity, sham 1 and sham 2 groups had significantly reduced the peripheral (A), central (B), and total (C) locomotor activity in comparison with the control group (*P*<0.001). Vit B12 (1 mg/kg), EB (50 mg/kg), either alone or in combination significantly improved peripheral (A), central (B), and total (C) locomotor activity compared with the sham groups (*P*<0.05, F (10, 66) = 70.92 (A), 10.97 (B), 17.32 (C), and *P*<0.001).

***Western blot analysis***

The effect of different doses of vit B12 (0.25, 0.5, and 1 mg/kg) and EB (25 and 50 mg/kg) on the rat hippocampus proteins is displayed in Figure 4. The results showed that the protein levels of non-phosphorylated AKT, BDNF, and CREB were significantly up-regulated, while the phosphorylated types of these proteins were down-regulated in the sham groups compared with the control group (*P*<0.05, *P*<0.01, and *P*<0.001). Vit B12 (1 mg/kg), EB (50 mg/kg), and their co-administration markedly decreased the non-phosphorylated AKT, BDNF, and CREB levels. However, the proteins levels of phosphorylated AKT, BDNF, and CREB increased statistically significant in comparison with the sham groups (*P*<0.05, *P*<0.01, F (5, 12) = 111.8, 157.7, and 302.3, and *P*<0.001).

## Discussion

The results of this study showed that vit B12 (1 mg/kg) and EB (50 mg/kg) either alone or in combination, at high doses, had a promoting effect on memory task or increased STL as well as peripheral, central, and total locomotor activity compared with the sham groups. Regulation of neurite outgrowth has a key role in neuronal plasticity and neuronal regeneration from injuries. It was clarified that the neuroprotective effect of vit B12 (1 mg/kg) and EB (50 mg/kg), especially in combination, was attributed to its activation of phosphorylated Akt/CREB signaling pathways through increasing the expression of phosphorylated BDNF. 

Although the precise mechanisms of demyelination have not been fully elucidated, several previous studies have shown that myelin destruction can happen via pro-inflammatory and neurotoxic factors produced by macrophages. For example, TNF-α and especially autoreactive CD4+ T cells can pass the blood-brain barrier, enter the central nervous system, identify the antigens of myelin sheaths, and start a chronic inflammatory cascade (33). The inflammatory environment in demyelinating MS lesions leads to the production of reactive oxygen species (ROS) and reactive nitrogen species (RNS), proinflammatory cytokines, specific demyelinating antibodies that contribute to disease development and progression (34). In fact, myelin destruction, astrocytic scar formation, and even axonal loss can be a major cause of long-term and permanent disability in patients with neurological disorders, such as Parkinson’s and MS diseases (35).

The hippocampus is involved in learning, memory, and attention processes through GABA and cholinergic receptors (28). Any damage resulting from internal or external factors, such as inflammation, can lead to cellular and molecular changes in synaptic integrity, axonal transport, and glutamate homeostasis (36) and impair learning and memory function in EAE mice (37) and MS patients (38). The most important ingredients for myelination and maintenance of neuron axons are vit B12, folic acid, and iron and their deficiency in serum and CSF cause myelin destruction and MS (39). 

In this study, we demonstrated that vit B12 reduced EtB-induced fear learning and memory, and locomotor activity impairments in a dose-dependent manner. As stated in previous studies, vit B12 has anti-inflammatory, neuroprotective, and immunomodulatory properties, such as a decrease in the activity of cytokines, like TNF-α (40, 41). In several studies, an important association has been reported between a decrease in the level of vit B12 and an increase in homocysteine concentration and demyelination (42). The vit B12 deficiency can result in damage to mitochondria, oxidative stress, apoptosis, cognitive dysfunction, and brain disorders, such as Alzheimer’s and MS diseases (41).

Estrogen, especially estradiol, via ER-β inhibits mitochondrial dysfunction against oxidative stress and apoptosis of neurons and protects neurons from demyelination (43)*. *Estriol and estradiol exert their anti-inflammatory effects through decreasing cytokines and chemokines (44) in the EAE model (45). There is a strong relationship between estrogens, hippocampal CA1 neurogenesis, and spatial learning and memory performances (46). Estradiol may have an effect on the dopaminergic system in the amygdala, prefrontal cortex, and nucleus accumbens (47). Dopamine is a central neurotransmitter that has an impact on effort-based decision-making (48).

According to previous studies, the results of this study showed that EB reduced EtB-induced fear learning and memory impairments and locomotor activity disorders at a dose-dependent manner. Although estradiol has a dual effect on learning and memory functions, some studies indicated that estrogen derivatives promoted learning and memory performances. It may be due to different methods, doses, time of exposure or even genomic and non-genomic effects (49). In one study estradiol administration into the dorsal hippocampus or prefrontal cortex facilitated or impaired the working memory, in the delayed win–shift test, depending on the dose and site of injection. The working memory was impaired 24 hr after estradiol injection into the hippocampus, but not the prefrontal cortex (49). Several studies have also expressed that a high level of estradiol impaired hippocampal-dependent spatial working/reference memory function in radial arm maze, caudate-dependent cued win-stay, and amygdala-dependent conditioned place preference (50), while low levels of estradiol facilitated working memory (50, 51).

We have also shown that vit B12 and EB alone or in combination therapy increased the phosphorylation of BDNF, CREB, and AKT proteins in a dose-dependent manner. BDNF, a small neuroprotective and anti-apoptotic secretory protein belongs to a class of the neurotrophin family (52). This protein plays a key role in survival and function of specific neuronal populations, including cholinergic and dopaminergic neurons, astrocytes, oligodendrocytes, and microglia cells, as well as reduction in nervous system immune response (52). The CREB activity has also a critical role in neuronal plasticity and memory formation in the brain. The CREB deficiency increases neurological disorders, such as Alzheimer’s disease (53, 54). In addition, Akt is an important signaling pathway for oligodendrocyte progenitor cell proliferation, differentiation, myelination, and memory formation (8, 20, 55). 

Estrogens trigger the expression of BDNF via BDNF receptor (trkB) (52). Steroid hormones, especially estrogen, also have a huge impact on the function of brain cells, particularly the cells that produce BDNF and trkB, including the olfactory bulb, hippocampus, cortex, amygdala, septum dorsolateral area of the bed nucleus terminals, and the lateral habenular nucleus (52). Reduced estrogen and BDNF levels were reported in patients with Parkinson’s disease and Alzheimer’s disease. In addition, estrogen and BDNF affect the function of cholinergic, GABA ergic, and dopaminergic neurons (56). Several studies have shown that estrogen depletion leads to decreased BDNF protein expression in the hippocampus and cortex regions in Alzheimer’s and Parkinson’s diseases (52). Estradiol also up-regulates the CREB protein and prevents apoptotic pathway (53, 54, 57). Numerous studies have found that ER-β ligand, including DPN (2,3-bis(4-hydroxyphenyl)-propionitrile) (20, 55, 58) and indazole chloride (8), reduced the clinical symptoms of the EAE disease. In addition, estrogen compounds exert their neuroprotective effect on progenitor’s proliferation, oligodendrocyte differentiation, remyelination, and locomotor function through enhancement of BDNF activity, phosphorylation of ER-β, and activation of PI3K/Akt/mTOR signaling pathway. The main goals in treatment of neurodegenerative diseases like MS are reducing the disease symptoms, preventing oligodendrocytes loss, and improving oligodendrocyte progenitor’s proliferation and differentiation. Another purpose is to identify an intervention strategy that stimulates endogenous remyelination and reduces disease progression. According to the findings of this study, it seems that vit B12 and EB have improved fear learning and memory, locomotor activity and increased phosphorylated Akt, BDNF and CREB proteins in rat hippocampus following EtB-induced demyelination in a dose dependent manner.

Con: Control; P: Peanut oil; D: Distilled water; Vit: Vitamin; EB: Estrogen benzoate; EtB: Ethidium bromide; STL: Step through latency (n=7)

**Figure 3:**
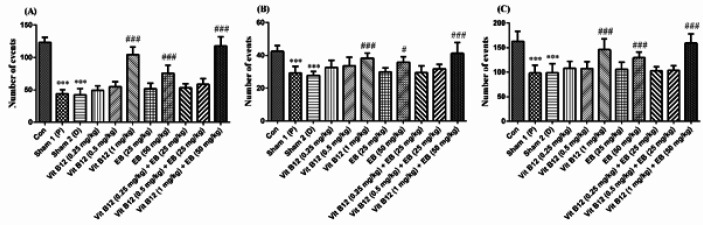
Effects of vit B12 (0.25, 0.5, and 1 mg/kg) and EB (25 and 50 mg/kg) alone or in combination therapy in peripheral (A), central (B), and total (C) locomotor activity. Animals received EtB in sterile 0.9% saline at the rate of 1 μl/min into the hippocampal CA1 area. Three days after injection, the rats were randomly divided into 11 groups. Distilled water (as vit B12 vehicle) and vit B12 were gavaged and peanut oil (as EB vehicle) and EB were administrated by intraperitoneal injection. The control group received intra-hippocampal saline (as solvent). Treatment duration was 5 days. Data are shown as mean±SD, one-way ANOVA and Tukey–Kramer. ^***^P<0.001 compared with the control group, and ^#^*P*<0.05 and ^###^*P*<0.001 compared with the sham groups

Con: Control; P: Peanut oil; D: Distilled water; Vit: Vitamin; EB: Estrogen benzoate; EtB: Ethidium bromide (n=7)

**Figure 4:**
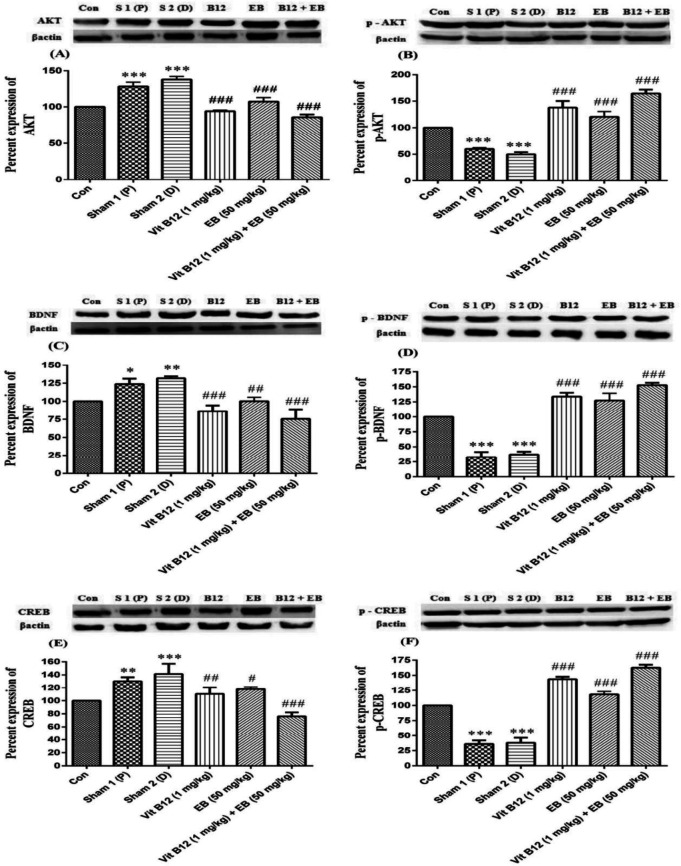
Effects of vit B12 (0.25, 0.5, and 1 mg/kg) and EB (25 and 50 mg/kg) alone or in combination therapy on the protein levels of AKT (A), p-AKT (B), BDNF (C), p-BDNF(D), CREB (E), and p-CREB (F). Animals received EtB in sterile 0.9% saline at the rate of 1 μl/min into the hippocampal CA1 area. Three days after injection, the rats were randomly divided into 11 groups. Distilled water (as vit B12 vehicle) and vit B12 were gavaged and peanut oil (as EB vehicle) and EB were administrated by intraperitoneal injection. The control group received intra-hippocampal saline (as solvent). Treatment duration was 5 days. Data are shown as mean±SD, one-way ANOVA and Tukey–Kramer. *P<0.05, **P<0.01, and ***P<0.001 compared with the control group and ^#^*P*<0.05, ^##^*P*<0.01, and ^###^*P*<0.001 compared with the sham groups

Con: Control; P: Peanut oil; D: Distilled water; Vit: Vitamin; EB: Estrogen benzoate; EtB: Ethidium bromide (n=7)

## Conclusion

According to the results of this study, cobalamin (1 mg/kg) and estradiol benzoate (50 mg/kg), either alone or in combination therapy significantly increased fear learning and memory, whereas they decreased the locomotor activities. Some portion of the effect of an exposure is mediated through up-regulating phosphorylated AKT, BDNF, and CREB in rat hippocampus. It seems that co-administration of cobalamin and estradiol benzoate can be helpful in treatment of MS. However, the discovery of the exact mechanisms of hormone effects on cognitive functions, proteins, and genes requires more comprehensive research and since high doses of estradiol often cause memory degradation, low doses of estradiol are recommended.
